# Cancer phenotype as the outcome of an evolutionary game between normal and malignant cells

**DOI:** 10.1038/sj.bjc.6605288

**Published:** 2009-09-01

**Authors:** D Dingli, F A C C Chalub, F C Santos, S Van Segbroeck, J M Pacheco

**Affiliations:** 1 Division of Hematology, College of Medicine, Mayo Clinic, Rochester, MN 55905, USA; 2 Departamento de Matemática da Universidade Nova de Lisboa and Centro de Matemática e Aplicações, Quinta da Torre 2829-516, Caparica, Portugal; 3 IRIDIA, CoDE, Université Libre de Bruxelles, Avenue Franklin Roosevelt 50, Brussels 1050, Belgium; 4 COMO, Vrije Universiteit Brussel, Pleinlaan 2, Brussels 1050, Belgium; 5 ATP-group, CFTC and Departamento de Fisica da Faculdade de Ciências, Lisboa Codex P-1649-003, Portugal

**Keywords:** somatic evolution of cancer, multiple myeloma, cancer dynamics, replicator cell dynamics, cancer ecology, evolutionary game theory of cancer

## Abstract

**Background:**

There is variability in the cancer phenotype across individuals: two patients with the same tumour may experience different disease life histories, resulting from genetic variation within the tumour and from the interaction between tumour and host. Until now, phenotypic variability has precluded a clear-cut identification of the fundamental characteristics of a given tumour type.

**Methods:**

Using multiple myeloma as an example, we apply the principles of evolutionary game theory to determine the fundamental characteristics that define the phenotypic variability of a tumour.

**Results:**

Tumour dynamics is determined by the frequency-dependent fitness of different cell populations, resulting from the benefits and costs accrued by each cell type in the presence of others. Our study shows how the phenotypic variability in multiple myeloma bone disease can be understood through the theoretical approach of a game that allows the identification of key genotypic features in a tumour and provides a natural explanation for phenotypic variability. This analysis also illustrates how complex biochemical signals can be translated into cell fitness that determines disease dynamics.

**Conclusion:**

The present paradigm is general and extends well beyond multiple myeloma, and even to non-neoplastic disorders. Furthermore, it provides a new perspective in dealing with cancer eradication. Instead of trying to kill all cancer cells, therapies should aim at reducing the fitness of malignant cells compared with normal cells, allowing natural selection to eradicate the tumour.

The origin of cancer requires the appearance of a new and *aberrant* cell type due to at least one mutation in a normal cell (Hanahan and Weinberg, 2000; Vogelstein and Kinzler, 2004). Cancer development is associated with the expansion of a mutant clone, and is normally described in terms of *frequency independent* evolutionary dynamics. Cancer cells have a different relative fitness compared with normal cells, leading to clonal expansion, *independent* of the relative abundance of different cell lineages. However, whenever one lineage expands, it often influences and is influenced by the current abundance of other cell populations (including normal cells). Such dynamic behaviour is best described in terms of *frequency dependent* selection (and mutation), akin to Evolutionary Game Theory (EGT) (Maynard-Smith, 1982; Hofbauer and Sigmund, 1998). Until now the applications of EGT to cancer have been mostly explorative in nature, without succeeding in providing insights into specific diseases (Tomlinson, 1997; Tomlinson and Bodmer, 1997; Axelrod *et al*, 2006). Here we demonstrate how detailed biochemical knowledge, accumulated over the last two decades, helps us in defining such games.

Let us consider the case of multiple myeloma (MM) bone disease, an important cause of morbidity due to pain, the risk of pathological fractures and neurological deficits. Bone loss may be focal (lytic lesions) (Berenson, 2001; Kyle and Rajkumar, 2004) or diffuse leading to osteoporosis (Harper and Weber, 1998). The appearance of MM cells alters (and is altered by) bone remodelling. *Normal* bone remodelling is a consequence of the dynamic balance between osteoclast (OC) mediated bone resorption and bone formation from osteoblast (OB) activity (Figure 1A), partly dependent on the receptor activator of nuclear factor-*κ*B (RANK), RANK ligand (RANKL) and osteoprotegerin (OPG) axis (Roodman, 2004; Terpos *et al*, 2007). The appearance and expansion of MM cells disrupts this dynamic equilibrium between OB and OC, with a disease progression time scale of the order of a few years (Kyle and Rajkumar, 2004). The presence of MM cells alters the OB–OC balance in favour of OC (Berenson, 2001; Roodman, 2004; Terpos *et al*, 2007). This process can be subdivided into two components:

- MM and stromal cells produce a variety of cytokines including interleukin 1*β* (IL-1*β*) (Dinarello, 2009a), RANKL (Croucher *et al*, 2001) and MIP-1*α* (Choi *et al*, 2000) (summarized as ‘osteoclast activating factors’ (OAF) (Roodman, 2004; Roux and Mariette, 2004)) that recruit OC precursors and stimulate growth of the OC population; and
- Secretion of Dickkopf-1 (DKK1) by myeloma cells directly inhibits Wnt3a regulated differentiation of osteoblasts, reduces OPG expression and alters the OPG–RANKL axis against OB activity (Roux and Mariette, 2004; Terpos *et al*, 2007; Qiang *et al*, 2008a, 2008b, 2008c).

The biochemical interactions between MM, OC and OB cells highlight the dependence of MM cells on the bone marrow microenvironment, at least early in the course of the disease (Kuehl and Bergsagel, 2002; Epstein and Yaccoby, 2003). As shown in Figure 1, IL-6 (Jelinek, 1999) and osteopontin (Abe *et al*, 2004), produced by OC cells stimulate growth of the MM cell population, hence conferring a *net* benefit to MM cells. On the other hand, production of OAF by MM cells (i) confers a *net* benefit to OC cells. Mechanism (ii) also leads to a potential disadvantage for OB cells in the presence of MM cells, whereas MM cells are unaffected by the presence of OB cells. Figure 1C provides a useful scheme to translate the complex exchange of chemical signals between different cell types into net benefits and costs of each cell type. We can therefore recast the frequency dependent balance between these cell populations in terms of an evolutionary game encompassing three ‘strategies’ – OC, OB and MM, and the following payoff matrix: 
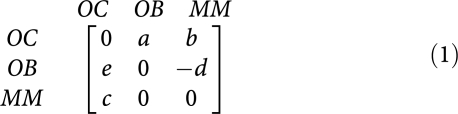


The present formulation is the mathematical equivalent of an ecosystem where the interactions between species (MM, OC and OB) are determined by matrix (1). Without loss of generality (see Materials and Methods) we assume that interactions between cells of the same type are neutral. In the absence of MM cells (*a*, *b*, *c*, *d* and *e* are non-negative), there is a stable balance between OC and OB cells with a OC-frequency given by *a*/(*e*+*a*) such that increasing *e* moves the equilibrium towards OB (Maynard-Smith, 1982). Hence, in the absence of MM cells, OB and OC engage in a co-existence game, the internal equilibrium of which reflects normal physiology. Any disturbance from this state induces an evolutionary dynamics that acts to restore the equilibrium (enabling normal tissue to adapt to changing demands and repair after injury). When we embed the OB–OC dynamics in a more general framework, including MM cells, the nature of the OB–OC equilibrium possibly changes from stable to a saddle point (for a certain set of parameters) or remains stable (otherwise). In the first case, even a small number of MM cells will be able to invade and disease will progress; in the second, the natural dynamics will prevent the system from the invasion of MM cells and the OB–OC equilibrium will be restored.

## Materials and methods

Our analysis is based on the replicator equation describing the frequency dependent evolutionary dynamics of three well-mixed cell populations (Hofbauer and Sigmund, 1998). Consequently, we assume that (i) no new mutations occur during tumour dynamics except those which initiated the tumour and (ii) deterministic cell population dynamics. Although the first issue is often justified (Traulsen *et al*, 2007; Dingli *et al*, 2008), cell dynamics often reflects a stochastic behaviour which may lead to additional disease variability not captured at this level of description. The generation of the first MM cell is a multistep process requiring a series of mutations that transform a normal plasma cell into an MM cell (Kuehl and Bergsagel, 2002; Bergsagel and Kuehl, 2005). We start our dynamics assuming that this first MM is present and follow its clonal expansion.

Tumour dynamics is conveniently represented in the simplex (an equilateral triangle for three strategies) at every point of which we have the relative frequencies of OB, OC and MM populations that sum up to 1. Let us denote by *x*_*i*_(*t*) the relative frequencies of the cell-types: *x*_1_(*t*) (OC cells), *x*_2_(*t*) (OB cells) and *x*_3_(*t*) (MM cells). The replicator equations read 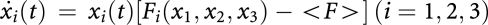 where the fitness of each cell type is given in terms of a game payoff matrix *A*_*ij*_ by 
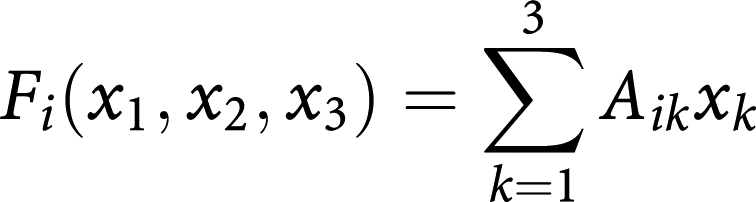
 whereas the average fitness of the population reads 
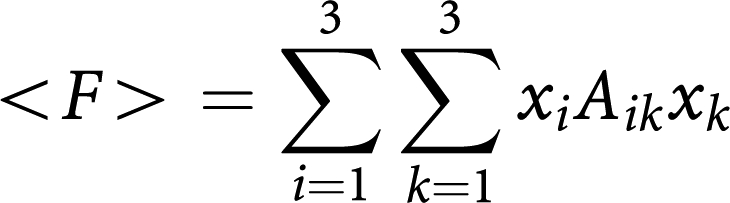


The replicator equations reflect a simple and intuitive dynamics for each cell type-depending on whether the fitness of a given cell type is higher (lower) than the average fitness of the entire population, that cell type will increase (decrease) in the total population at a rate specified by the replicator equations.

The benefits and costs resulting from interacting cell populations are captured in the initial payoff matrix (1) here associated with matrix *A*_*ij*_. We reduce this matrix to the minimal payoff matrix 
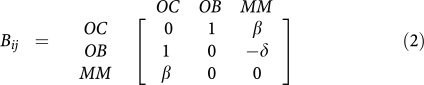
 by taking into account that the nature of fixed points of evolutionary dynamics (though *not* their location) remains unaffected under a projective transformation of the relative cells frequencies (Hofbauer and Sigmund, 1998). In the present case, the matrices are related by 
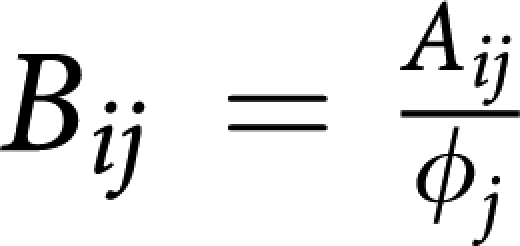
 where *ϕ*_*i*_ are positive constants given by (*ϕ*_1_*,ϕ*_2_*,ϕ*_3_)=(*e,a,be/c*), such that 
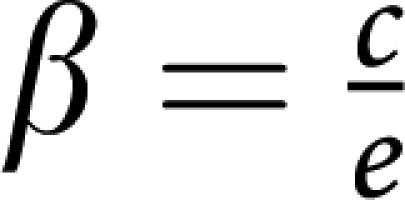
 and 
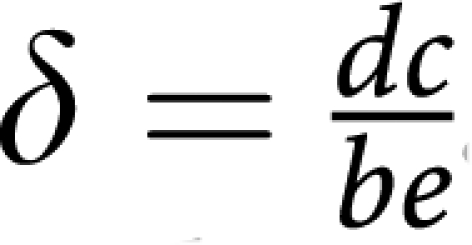
. The matrix transformation may be shown to correspond to the following projective transformation of the relative frequencies *x*_*i*_ → *y*_*i*_ (Hofbauer and Sigmund, 1998): 
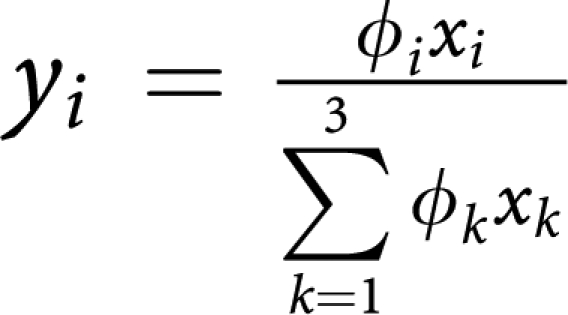
 which, by rescaling the relative frequencies, changes the location of the fixed points on the simplex, without changing their stability nature. Note further, that the replicator equations and associated dynamics remain unaffected if we add an arbitrary constant to each column of the payoff matrix. In other words, it is always possible to zero all the diagonal elements of the game payoff matrix.

The fixed points (x_1_^*^,x_2_^*^,x_3_^*^ of the evolutionary dynamics under matrix *B*_*ij*_ are readily found. Two vertices of the simplex, (0,1,0) and (1,0,0) are unstable fixed points, whereas the third is a saddle point. The fixed point (½,½,0) associated with normal physiology is unstable whenever *β*>1, being stable otherwise; the fixed point (½,0, ½) is a stable fixed point whenever *β*>1 or whenever *β*<1 but *β*+*δ*>1; in this last situation, a saddle point arises in the interior of the simplex, located at 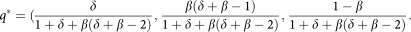


Indeed, we easily prove the following

**Theorem**: q^*^ is in the interior of the simplex if *β*<1 and *β*+*δ*>1.

**Proof**: q^*^ is in the interior of the simplex if 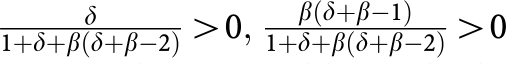 and 
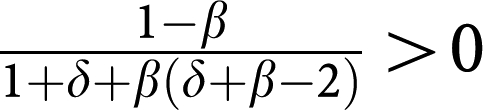


. The first two conditions readily imply that *β*+*δ*>1, whereas the third one is equivalent to demand that *β*<1. ▪

Furthermore, we also prove that

**Theorem**: If q^*^ is in the interior of the simplex, then it is a saddle.

**Proof**: The eigen value associated with q^*^ reads 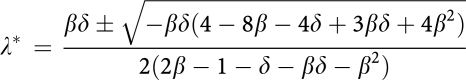


As 3β^2^δ^2^< − βδ(4 − 8β − 4δ + 3βδ + 4β^2^), this means that the first eigen value is positive and the second is negative. ▪

In our framework, additional mutations within the expanding MM population can act to effectively change the values of the interacting parameters *β* and/or *δ*; in general, however, we expect that such modifications will contribute in changing the time scale of the dynamics, but not their nature or the final outcome. Similarly, if we consider cytotoxic chemotherapy that significantly reduces the MM population, this will act to reset the clock and, in some cases, significantly increase life expectancy, but will not alter the final outcome (i.e., the equilibrium). This is supported by ample clinical data, whereby high-dose chemotherapy and stem cell transplantation lead to significant reductions in tumour burden with an improvement in survival but the disease invariably relapses (Attal *et al*, 1996, 2003).

## Results

Figure 2 provides an overview of the possible scenarios that emerge from the evolutionary dynamics associated with payoff matrix (2). The three strategy game leads to unstable equilibria whenever the population is monotypic: the presence of a single cell of any of the other type leads to the co-existence of two strategies, as the pair-wise games OB–OC and OC–MM are co-existence games (Maynard-Smith, 1982). The number and nature of the fixed points in the simplex will depend on the relative balance between *β* and *δ* in the payoff matrix, as shown in Figure 2 (and Materials and Methods).

If the net benefit that OC cells obtain from MM cells is smaller than what they get from OB cells (*β*<1), the population of MM cells can get extinct, and OB and OC may re-establish the stable dynamic equilibrium (Figure 2A). However, various studies suggest that *β*<1 is the exception rather than the norm (Roodman, 2002; Epstein and Yaccoby, 2003). Whenever *β*>1, the only stable equilibrium is the co-existence of MM and OC cells (Figures 2C, D and 3). In this extreme situation a part of bone is completely devoid of OB and as the bone approaches this state it is at increasing risk of fracture, a common feature of MM (Bataille and Harousseau, 1997; Kyle and Rajkumar, 2004). One need not postulate a negative effect of MM on OB: even if *δ*=0 (MM and OB are neutral with respect to each other), the only stable equilibrium is the co-existence of MM and OC (Figure 2C). However, changes in *δ* may have a significant effect on the life history of the disease and associated progression time, as shown in Figure 2D: Increasing *δ* for fixed *β* – that is, increasing the disadvantage of OB cells in the presence of MM cells – leads to disease dynamics, in which considerable bone loss occurs without a significant increase in the MM population. This may explain instances of myeloma-induced osteoporosis without a massive MM cell burden. Similarly, increasing *β* leads to more bone destruction, higher tumour burden and faster tumour progression. This behaviour is observed clinically, patients with higher MIP-1*α* levels (increasing *β*) tend to have more bone resorption and lytic bone lesions (Terpos *et al*, 2003; Hashimoto *et al*, 2004) and shorter survival due to a higher tumour burden (Terpos *et al*, 2003). Consequently, therapies which suppress or reduce *β* (inhibiting, e.g., MIP-1*α* secretion or IL-1*β* (Lust *et al*, 2009; Dinarello, 2009b)) should decrease the number of lytic lesions and the speed of disease progression, prolonging survival (Choi *et al*, 2001). Similarly, any therapy that decreases *δ* (e.g., Dkk-1), should reduce the myeloma burden, slow the progression of the disease, and improve bone mass (Yaccoby *et al*, 2007).

We also used the model to understand the effect of high-dose therapy and stem cell transplantation that is often offered to patients with myeloma. This therapy normally leads to approximately 3 log reduction in tumour burden (Dingli *et al*, 2007a, 2007b). Patients going to transplant have variable tumour burden, so we evaluated the effect of initial tumour burden and depth of response on bone and tumour cell population dynamics (Figure 4). Across a wide range of parameters, we find that bone healing does not occur, that is the ratio of OB : OC does not return to normal as long as MM cell persists (Figure 4B, D and E). As the depth of response increases, the time-to-relapse increases (Figure 4B vs D). Moreover, the speed of relapse depends on both the MM population at the time of transplant as well as the degree of tumour cytoreduction achieved with transplantation.

**Figure 1:**
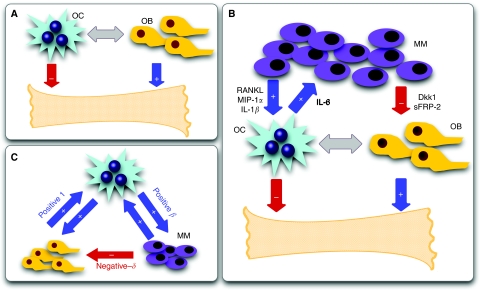
Bone turnover under normal and pathological conditions. (**A**) Normal bone remodelling reflects the balance between osteoclasts (OC) that resorb bone and osteoblasts (OB) that regenerate bone. (**B**) The presence of multiple myeloma cells (MM) alters bone homeostasis by cytokine production (e.g., interleukin (IL)-1*β*, receptor activator of nuclear factor-*κ*B ligand (RANKL) and MIP-1*α*) that recruit and activate OC, increasing resorption. OC may also produce growth factors (IL-6) for MM cells, which may also secrete cytokines that suppress OB activity. The synergy between MM and OC confers them an advantage with respect to OB. (**C**) Cytokine exchanges between cells lead to net effects associated with payoff matrix entries.

The payoff matrix (2) provides a minimal description of the disease and identifies the kernel features which determine tumour behaviour and dynamics. It is important to stress that the non-trivial identification of the key parameters *β* and *δ* could not be anticipated from the rationale underlying the model setup, as illustrated in Figure 1 and out of which matrix (1) was derived. Instead, it results from a rigorous mathematical property of evolutionary game dynamics, which dictates that the evolutionary outcome of the disease can be equivalently analyzed in terms of matrix (2). Under the present assumptions, *β* and *δ* are sufficient to characterize myeloma bone disease. However, it is well known that the natural history of the disease is variable, presumably due to genetic and epigenetic differences in myeloma cells from different patients. Normal physiology also varies from individual to individual. The payoff matrix (1) takes this variability into account by incorporating those effects, which do not relate directly to MM disease (e.g., pre-existing disease that could alter the tumour–host interactions) but can affect the overall disease dynamics. Figure 3 shows how different entries in payoff matrix (1) sensitively affect disease progression *paths* and *times*. We assumed that the OB–OC equilibrium does not change qualitatively from patient to patient, as opposed to what one may expect concerning the OC–MM final equilibrium. The actual values of payoff matrix (1) affect both the location of the fixed points and the time associated with disease progression. These are expected to be patient specific, reflecting tumour–host interactions and variability of disease due to differences in the host rather than the malignant cell genotype.

## Discussion

**Figure 2:**
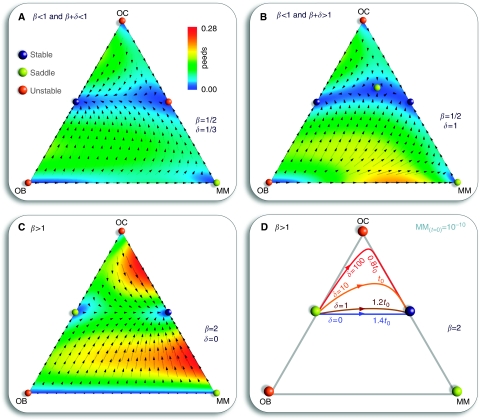
Evolutionary dynamics of osteoclasts (OC), osteoblasts (OB) and multiple myeloma (MM) cell types. Vertices mean that cell population is monotypic. Bone homeostasis occurs in the absence of MM (OC–OB line), remaining stable in the presence of MM if *β*<1 (upper panels). The fixed point along OC–MM is unstable whenever *β*+*δ*<1, (**A**) being stable otherwise. (**B**) For *β*>1 only the OC-MM co-existence equilibrium is stable (lower panels). (**C**) Colour gradients represent the rate of disease progression. (**D**) *δ* affects both the path *and* progression time between OB–OC and OC–MM equilibrium. OB–OC equilibrium was disturbed by introducing one MM cell (in a population of 10^10^ cells), for different values of *δ*.

Besides giving a broad view of the overall features of the disease (captured by *β*>1 and *δ*⩾0), our approach provides important insights on therapeutic priorities to ameliorate the patient's condition. Therapies that kill MM cells can slow the speed of disease evolution and prolong patient survival, while simultaneously improving bone structure. Unfortunately, this will not alter the final outcome. Indeed high-dose chemotherapy and stem cell transplantation, the current ‘standard of care’ for eligible patients, lead to significant reductions in tumour burden with an improvement in survival but the disease invariably relapses and many patients ultimately die of relapsed or refractory disease (Attal *et al*, 1996, 2003). In contrast, agents such as bisphosphonates can change disease dynamics by altering the ‘game’ (reducing *β*) and consequently reduce the number of lytic bone lesions or myeloma-induced osteoporosis. Whenever therapies succeed in reversing the sign of (*β*−1), they may inhibit disease progression. For example, antisense therapy against MIP-1*α* blocked bone destruction in a mouse model of myeloma (Choi *et al*, 2001). Similarly, therapies that reduce the effective value of *δ* may significantly attenuate morbidity by slowing the speed of bone loss. In this respect, an antibody directed against *Dkk-1* reduced both bone loss and the myeloma tumour burden in a preclinical model (Yaccoby *et al*, 2007). This general behaviour is also supported by data from a human clinical trial. A recent study by Lust *et al* (2009) showed that neutralization of IL-1*β* (an osteoclast activating factor that also stimulates IL-6 production) with an IL-1 receptor antagonist (IL-1Ra, known as anakinra), slows the progression of smouldering to active multiple myeloma. This finding can be easily understood in light of the present model: anakinra effectively reduces *β*, which has precisely the same implications regarding disease progression as shown in Figure 2. Patients treated with this agent had a reduction in the rate of proliferation of plasma cells and accumulation of lytic bone lesions (Lust *et al*, 2009; Dinarello, 2009b).

It is worth stressing the fact that whenever *β*<1 and *β*+*δ*>1 (Figure 2B), the appearance of an interior fixed point may be of particular relevance in what concerns the development of new therapies. Indeed, our model predicts that by altering the relative fitness of one cell type with respect to the others, one may effectively change the overall disease evolution, in this case to the appearance of such an internal saddle point. Thus, our model suggests that therapies directed against *Dkk-1* may be useful in reducing bone loss and in slowing down the accumulation of tumour burden in patients with smouldering myeloma. It is therefore important to determine at which stage of disease evolution such a therapeutic procedure is implemented. Therapies that bring the disease into this regime may be effective in patients, that at the time of diagnosis are in a disease state that is metaphorically located ‘to the left’ of the saddle point, in which case evolutionary dynamics will naturally lead to the decline of the MM cell population. The location of this saddle point is determined in part by the detailed interactions of the cell populations with each other. In our model we do not consider the possible existence of various subpopulations of MM cells that are dependent on the marrow microenvironment to variable extents and for which this model may or may not apply. If experimental data describing the interaction of such MM subpopulations (e.g., myeloma stem cells) become available, this can be incorporated into the model at the expense of increased mathematical complexity.

**Figure 3:**
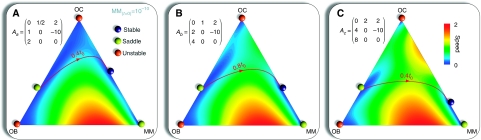
Host-specific tumour progression. The values *b* and *c* in matrix (1) alter the position of the equilibrium on the osteoclasts (OC) to multiple myeloma (MM) line (**A, B, C**), whereas *a* and *e* change the equilibrium between OC and osteoblasts (OB) cells, producing deviations both in the disease path and on the characteristic evolutionary time scale. Each panel (**A**, **B** and **C**) shows a different path with the associated matrix, all leading to the same payoff matrix (2) with *δ*=10 and *β*=2. We start with a small perturbation of the OC–OB equilibrium and *t*_0_ stands for the progression time of the configuration *δ*=10 and *β*=2 in payoff matrix (2) (Figure 2D).

**Figure 4:**
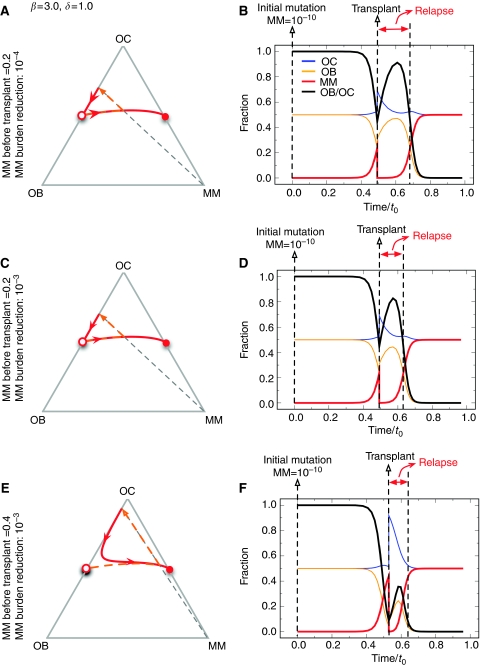
Population dynamics in response to therapy. Starting with different fractions of multiple myeloma (MM) cells (**A**, **C** and **E**), we determined the population trajectories as a function of reduction in MM burden (**B**, **D** and **F**). The general behaviour did not change across a wide range of parameter values. However, the speed of relapse is related to the starting size of the MM population. Note that the OB : OC ratio does not return to normal as long as MM cells persist.

Finally, there is variability in the precise location of the fixed points and the different possible *paths* that join them. One should not overlook the message from Figures 2D and 3 – the variability in tumour genotype/phenotype determines a corresponding variability of the time scales and life histories associated with disease progression. Our model illustrates how interactions between the genotype of normal and neoplastic cell populations, combined with the individual host specificities, determine both the phenotype and the dynamics of the disease, including disease progression times. As shown in Figure 2D, the nature of the interaction between MM and OB cells, controlled by *δ*, plays a very crucial role in both disease progression and expression. Patients with a disease characterised by a large *δ* will experience insidious disease with a small population of MM cells leading to significant and rapid bone loss, a feature that could lead to a misdiagnosis of osteoporosis rather than myeloma. In this era of individualised medicine, every patient is a special case and it is crucial to understand the tumour and host characteristics to better understand the patient's unique disease dynamics.

The model can be used to evaluate the potential for bone healing as the MM burden is reduced. At present, there are no therapeutic options that can cure the disease, so elimination of the whole MM population is not possible. As the MM cell burden is reduced, one expects healing of bone and increased life expectancy of the patient. However, the model predicts that bone healing would be a slow process and, given the low likelihood of eliminating all the MM cells relapse will take place, except perhaps by development of therapies that effectively change *β* and *δ*.

In the current model we consider tumour burden as the only parameter that has an effect on survival. Although patients with multiple myeloma can also die because of infection, renal failure or unrelated causes, there is a clearly defined relationship between tumour burden and mortality. Indeed, both the Durie–Salmon staging system and the more recent International Staging System depend directly or indirectly on an estimation of the tumour burden to predict survival (Durie and Salmon, 1975; Greipp *et al*, 2005).

The approach developed here is general and readily applicable to other diseases. Furthermore, it provides a new paradigm for dealing with cancer eradication: Instead of trying to kill all cancer cells, therapies should aim at changing the rules of engagement between different cell types, in our case parameters *β* and *δ*. In the mathematical language of EGT, this means that therapies should aim at literally *changing the dynamics*, enabling normal cells to out-compete the malignant clone, consequently leading to its evolutionary extinction.
